# CH-π Interaction Driven Macroscopic Property Transition on Smart Polymer Surface

**DOI:** 10.1038/srep15742

**Published:** 2015-10-29

**Authors:** Minmin Li, Guangyan Qing, Yuting Xiong, Yuekun Lai, Taolei Sun

**Affiliations:** 1 State Key Laboratory of Advanced Technology for Materials Synthesis and Processing, Wuhan University of Technology, 122 Luoshi Road, Wuhan 430070, P. R. China; 2 School of Chemistry, Chemical Engineering and Life Science, Wuhan University of Technology, 122 Luoshi Road, Wuhan 430070, P. R. China; 3 College of Textile and Clothing Engineering, Soochow University, 199 Ren’ai Road, Suzhou 215123, P. R. China

## Abstract

Life systems have evolved to utilize weak noncovalent interactions, particularly CH-π interaction, to achieve various biofunctions, for example cellular communication, immune response, and protein folding. However, for artificial materials, it remains a great challenge to recognize such weak interaction, further transform it into tunable macroscopic properties and realize special functions. Here we integrate monosaccharide-based CH-π receptor capable of recognizing aromatic peptides into a smart polymer with three-component “Recognition-Mediating-Function” design, and report the CH-π interaction driven surface property switching on smart polymer film, including wettability, adhesion, viscoelasticity and stiffness. Detailed studies indicate that, the CH-π interaction induces the complexation between saccharide unit and aromatic peptide, which breaks the initial amphiphilic balance of the polymer network, resulting in contraction-swelling conformational transition for polymer chains and subsequent dramatic switching in surface properties. This work not only presents a new approach to control the surface property of materials, but also points to a broader research prospect on CH-π interaction at a macroscopic level.

Creating new materials by taking inspirations from biological systems represents one of major challenges for materials science in 21st century^1^,^2^. Recently, enormous efforts have been made to design and develop bioactive materials with excellent biological responsiveness and tunable macroscopic properties or functions, because of their attractive application prospect in various biochemical devices, such as bio-sensors^3^,^4^, bio-actuators^5^,^6^, microfluidic channels^7^. An appealing strategy for designing such smart materials is to mimic the life systems, as typical examples, DNA or proteins usually adopt highly cooperative multiple weak interactions (e.g., hydrogen bonding, π-π stacking, hydrophobic interaction) to regulate highly ordered self-assembly, complexation or dissociation with ligands, and perform various biofunctions^8^,^9^,^10^,^11^. To achieve this bioinspired strategy, one of key issues is to connect these weak interactions with macroscopic properties of materials^12^. In this respect, smart polymers, particularly those based on poly(*N*-isopropylacrylamide) (PNIPAAm) system, provide an ideal platform to tackle this problem^13^,^14^, due to their excellent macroscopic response to weak interaction signals stemming from biomolecules recognition events by incorporating recognition receptors into the polymer systems^15^,^16^,^17^,^18^.

On the other hand, as a weak interaction occurring between carbohydrate and protein (Fig. 1a)^19^,^20^,^21^, CH-π interaction has been recognized to play important roles in cellular communication^22^, immune response^23^, and protein folding^24^. However, due to the weak affinity, most experimental and theoretical studies on CH-π interaction were confined to the single molecular level, e.g., host-guest recognition^25^,^26^, evaluation of energetic scale^27^,^28^, investigation of detail nature^29^,^30^. And it remains a challenge to extend such study to a broader level, for example, utilizing CH-π interaction to regulate macroscopic property of materials and further realize special functions. Taking advantage of the “Recognition-Mediating-Function” (R-M-F) design concept for smart polymer^31^,^32^, here we integrate monosaccharide-based CH-π receptor capable of recognizing aromatic peptide into a smart polymer, and report the CH-π interaction driven surface property transition on such polymer surface. In this design, monosaccharide-based CH-π receptor (Glc) works as a recognition unit, trifluoromethyl phenylthiourea component (PT) serves as a mediating unit, both of which were randomly copolymerized into PNIPAAm function main chain (PNI) to generate a grafted copolymer on solid substrate (denoted as PNI-*co*-PT-*co*-Glc) (Fig. 1b). Such a copolymer is not a mere copolymer consist of three units, whereas these units interact and cooperatively involve in the formation and alteration of copolymer conformation. After the copolymer film was treated by model peptides (Fig. 1c), an intriguing phenomenon was observed: relative hydrophobic peptides composed of aromatic amino acids, e.g., FFF (Phe-Phe-Phe), caused significantly larger changes in surface wettability, adhesion, viscoelasticity and stiffness than hydrophilic peptides (Fig. 1d). Mechanism studies indicated that, CH-π interaction between saccharide unit in copolymer chain and aromatic peptide induced complexation of them, which broke the initial amphiphilic balance of the copolymer network, resulting in contraction-swelling conformational transformation for copolymer chains and subsequent macroscopic surface properties transition. This work not only presents a new strategy to regulate the surface property of materials, but also points to a broader research prospect on CH-π interaction at a macroscopic level.

## Results and Discussion

**Figure 1:**
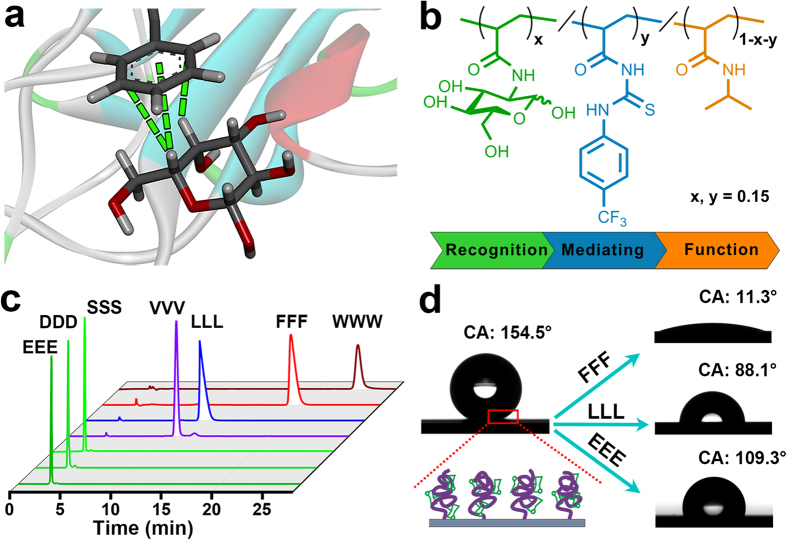
The overall scheme of this work. (**a**) The typical CH-π interaction identified in a galactose-lectin complex (PDB ID: 1AX1)^21^. (**b**) Molecular structure of the three-component PNI-*co*-PT-*co*-Glc copolymer. (**c**) Relative hydrophobicity scales of seven model tripeptides characterized by retention time in HPLC with C18 column. (**d**) Profiles of water droplet on the PNI-*co*-PT-*co*-Glc copolymer films on textured silicon substrate before and after being treated by different tripeptides.

In this work, we chose PNIPAAm as functional framework to construct smart copolymer. And, monosaccharide unit Glc was chosen as CH-π interaction receptor capable of recognizing and binding aromatic peptides. Moreover, trifluoromethyl phenylthiourea component (PT) was introduced as a mediating unit for regulating the surface hydrophobicity in order to obtain more substantial wettability change. On the basic of this design, the three-component random copolymer PNI-*co*-PT-*co*-Glc was firstly grafted onto flat silicon substrate by surface initiated atom transfer radical polymerization (see Methods section for details)^33^, and the acquired film was about 33 nm in thickness. In addition, in order to assess the impact on surface property of the film upon interaction with peptides, a set of tripeptides with different hydrophobicity scales was synthesized as model peptides (see Supplementary information for purity and identification data). Here, EEE (Glu-Glu-Glu), DDD (Asp-Asp-Asp) and SSS (Ser-Ser-Ser) were chosen as highly hydrophilic peptides, VVV (Val-Val-Val) and LLL (Leu-Leu-Leu) represented the peptides with moderate hydrophilicity, while FFF and WWW (Trp-Trp-Trp) were relative hydrophobic peptides due to their bulky aromatic rings, as determined by retention time in high-performance liquid chromatography (HPLC) (Fig. 1c and Supplementary Fig. S6).

**Figure 2:**
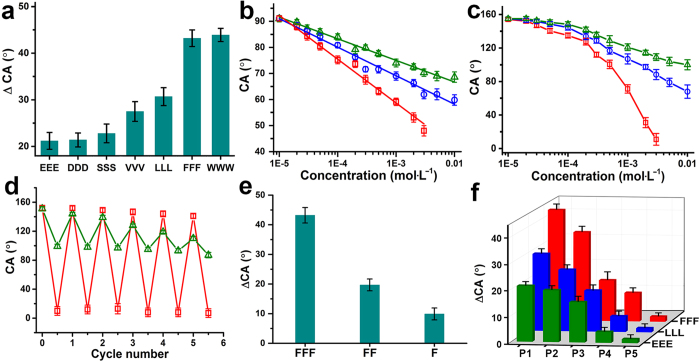
Peptide-selective wettability changes on the PNI-*co*-PT-*co*-Glc copolymer film. (**a**) Static contact angle decrease (ΔCA) after the film on flat silicon substrate was treated by different tripeptide solutions (3 mmol∙L^−1^). (**b**,**c**) Relationships between CAs of the film on a flat (**b**) or a textured silicon substrate (**c**) and tripeptide concentrations. Red: FFF; Blue: LLL; Green: EEE. (**d**) Cycling experiment for alternately treating the film on textured silicon substrate by tripeptide solutions (3 mmol∙L^−1^) and pure water. Red: FFF; Blue: LLL; Green: EEE. (**e**) ΔCAs of the PNI-*co*-PT-*co*-Glc copolymer film on flat silicon substrate treated by FFF, FF and F solutions (3 mmol∙L^−1^), respectively. (**f**) Comparison of ΔCAs on different polymer films on flat silicon substrate treated by tripeptide solutions (3 mmol∙L^−1^), respectively. P1: PNI-*co*-PT-*co*-Glc, P2: PNI-*co*-PT-*co*-AcGlc, P3: PNI-*co*-PT, P4: PNI-*co*-Glc, P5: PNIPAAm. All data are shown as mean ± s.d. (n = 4 ~ 5).

Water contact angle (CA) measurements were adopted to study the wettability responses of the PNI-*co*-PT-*co*-Glc copolymer film to various tripeptides^34^. The as-prepared film is hydrophobic originally with a static CA (apparent CA) of 92.2 ± 1.4°. After being immersed in each tripeptide aqueous solution at an equal concentration of 3 mmol∙L^−1^ for 10 min, followed by removal of any remaining excess liquid and a subsequent drying process by nitrogen gas flow, the films became hydrophilic and obvious CA decreases (ΔCAs) were observed (Fig. 2a). And the CA of the film could revert to the original value after further treatment by pure water. Interestingly, FFF and WWW caused much larger ΔCAs (43.7 ± 2.3° and 44.3 ± 1.9°, respectively) than LLL (31.3 ± 1.8°) and VVV (28.2 ± 2.4°) for the copolymer film surface. Moreover, the ΔCAs were only 21.1 ± 1.3°, 21.3 ± 2.0° and 22.3 ± 2.5° when SSS, DDD and EEE were evaluated, respectively. This phenomenon was further validated by the linear relationships between CA values and logarithms of tripeptide concentration (Fig. 2b and Supplementary Fig. S7). The slope for FFF is −16.8/log10, which is numerically larger than those of LLL (−11.1/log10) and EEE (−8.7/log10). Meanwhile, dynamic CA measurements also gave a consistent result (see Supplementary Fig. S8).

The PNI-*co*-PT-*co*-Glc copolymer film was also prepared on textured silicon substrate composed of well-aligned micropillars and nanofibrous structures on top of them (see Supplementary information for detailed preparation procedure of textured silicon substrate). Because of the amplification effect of surface micro- and nanostructures on wettability^35^, the film is superhydrophobic originally with a CA of 154.5 ± 2.2°. Figure 2c shows the concentration dependent curves of CA on this film after being treated by tripeptide solutions. For FFF, the CA decreased dramatically with the increase of concentration, reaching a value of 11.3 ± 3.6° at 3 mmol∙L^−1^. Nevertheless, for LLL and EEE, the CAs decreased slightly and finally gave the values of 88.1 ± 2.7° and 109.3 ± 2.4° at a higher concentration of 10 mmol∙L^−1^, respectively. These results show a peptide-selective wettability switching: relative hydrophobic tripeptides (e.g., FFF) induced much more obvious wettability change than hydrophilic ones (e.g., EEE) with a maximum ΔCA of nearly 100°, which could be discriminated directly by water droplet profiles (Fig. 1d). Cycling experiments for alternately treating the film by tripeptide solutions (FFF, LLL and EEE) at a concentration of 3 mmol∙L^−1^ and pure water further proved the significance and reliability of this effect (Fig. 2d). All comparisons in CA were conducted under the condition of aqueous solution with the equal concentration to exclude the possibility of wettability change caused by relative solubility of tripeptides. Therefore, considering the difference among tripeptides in chemical structures, we presume that aromatic rings of relative hydrophobic tripeptides may play critical roles in this process, which deserves an in-depth investigation.

To address this point, tetrapeptide FFFF (Phe-Phe-Phe-Phe), dipeptide FF (Phe-Phe) and F (Phenylalanine) were introduced to perform a control experiment. Compared with the obvious ΔCA (43.7 ± 2.3°) caused by FFF, under the same concentration, the ΔCAs were only 19.2 ± 1.6° and 10.6 ± 1.2° after the PNI-*co*-PT-*co*-Glc copolymer films on flat silicon substrate were treated by FF and F solutions, respectively, as shown in Fig. 2e. However, FFFF still caused a ΔCA of 34.3 ± 2.0° (see Supplementary Fig. S9). This reveals that, from the perspective of peptides, the number of aromatic groups in a peptide is closely related to wettability response of the PNI-*co*-PT-*co*-Glc copolymer film, and tripeptide should be the optimal choice for this study.

In order to verify the rationality of R-M-F design, three reference polymer films on flat silicon substrate, namely PNIPAAm, PNI-*co*-Glc, PNI-*co*-PT were prepared, and the wettability responses of which were investigated using tripeptide FFF, LLL, and EEE solution at an equal concentration of 3 mmol∙L^−1^. The original CA for PNIPAAm, PNI-*co*-Glc, PNI-*co*-PT and PNI-*co*-PT-*co*-Glc film was 61.1 ± 2.0°, 64.7 ± 1.9°, 104.5 ± 1.2° and 92.2 ± 1.4°, respectively. Therefore, this difference indicate that the presence of PT units obviously enhanced the initial surface hydrophobicity. On the other hand, the pure PNIPAAm film almost did not exhibit any evidential ΔCA after being treated by FFF, LLL, and EEE (P5, Fig. 2f), implying that the wettability response was not induced by physical adsorption of tripeptides. For the PNI-*co*-PT film, although ΔCAs were remarkable, no obvious difference could be observed (P3, Fig. 2f). By comparison, distinct difference in ΔCAs was observed for the PNI-*co*-Glc film (P4, Fig. 2f), but the ΔCA values were smaller than those on the PNI-*co*-PT film, due to the absence of the PT units. Therefore, these results indicate that the monosaccharide Glc units indeed serve as recognition units capable of discriminating the aromatic peptides from other peptides, and interaction between Glc and atomatic peptides is the main driving force for the responsive wettability switching. Moreover, the participation of PT units significantly enlarge the extent of surface wettability switching (see Supplementary Table S2 for detailed data). In addition, a PNI-*co*-PT-*co*-AcGlc copolymer film (AcGlc denotes the tetra-acetylated monosaccharide unit) on flat silicon substrate was also prepared, in which the OH groups were acetylated to minimize the influence of hydrogen-bonding (H-bonding). Interestingly, the absence of OH groups only had a subtle effect on the wettability response, the treatment of FFF still caused a larger ΔCA (36.2 ± 1.8°) than those of LLL (23.5 ± 1.7°) and EEE (19.3 ± 1.5°) (P2, Fig. 2f). This indicates that CH groups rather than OH groups in monosaccharide play a crucial role in the binding between monosaccharide unit in copolymer chain and aromatic group in FFF. This point is in accordance with the typical characteristics of CH-π interaction^36^,^37^.

**Figure 3:**
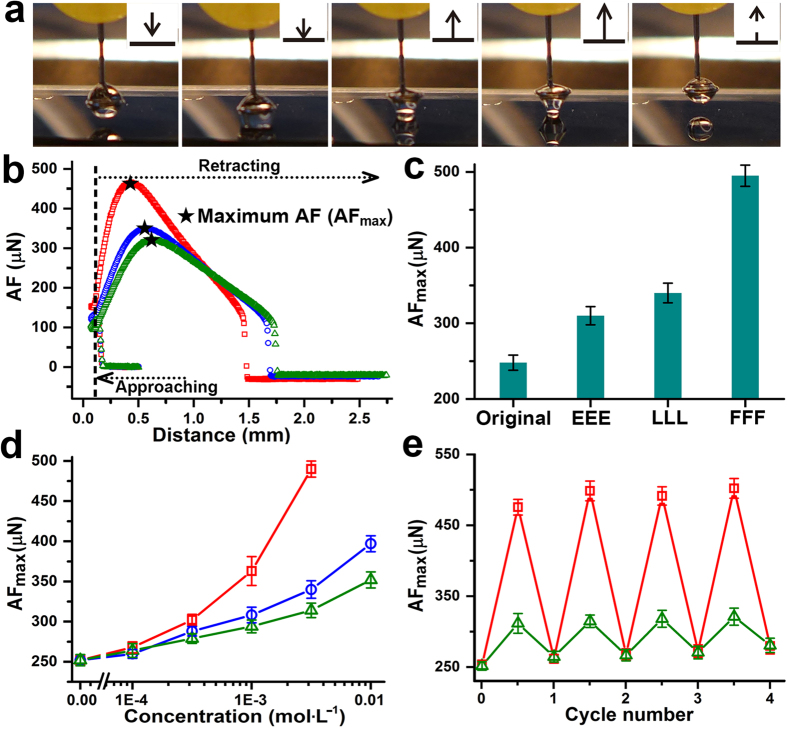
Peptide-selective adhesion force (AF) changes on the PNI-*co*-PT-*co*-Glc copolymer film on flat silicon substrate. (**a**) Graphical illustration of a typical AF measurement process. Insets show relative movements and shape changes of the water droplet. Dynamic AF curves (**b**) of the film treated by tripeptide solutions (3 mmol∙L^−1^), respectively, and the comparison of the maximum AFs (**c**). Red: FFF; Blue: LLL; Green: EEE. (**d**) Relationships between the maximum AFs and tripeptide concentrations. Red: FFF; Blue: LLL; Green: EEE. (**e**) Cycling experiments for alternately treating the film by tripeptide solutions (3 mmol∙L^−1^) and pure water. Red: FFF; Green: EEE. All data are shown as mean ± s.d. (n = 3 ~ 5).

As another important surface property closely related to wettability^38^, water adhesion of the PNI-*co*-PT-*co*-Glc copolymer film on flat silicon substrate in response to different tripeptides was investigated using high-sensitivity microelectromechanical balance system. Figure 3a illustrates the detailed process of water adhesive force (AF) measurement on the film. After the film was treated by tripeptide solution through the same procedure adopted in CA measurements, the surface adhesive force was measured accurately and the distinct adhesive force curves were obtained, as shown in Fig. 3b. A maximum AF of 495 ± 8 μN was obtained on the film after FFF treatment, which was much larger than those for LLL (330 ± 10 μN) and EEE (304 ± 9 μN) (tripeptide concentration: 3 mmol∙L^−1^) (Fig. 3c). The relationships between AFs and tripeptide concentrations further proved this difference (Fig. 3d). In addition, the AF could revert to the original value, which also exhibit good reversibility upon alternate treatments by tripeptide solutions and pure water (Fig. 3e). These results are highly consistent with those in CA measurement, and further solidify the effect of CH-π interaction from the perspective of copolymer surface adhesion switching.

**Figure 4:**
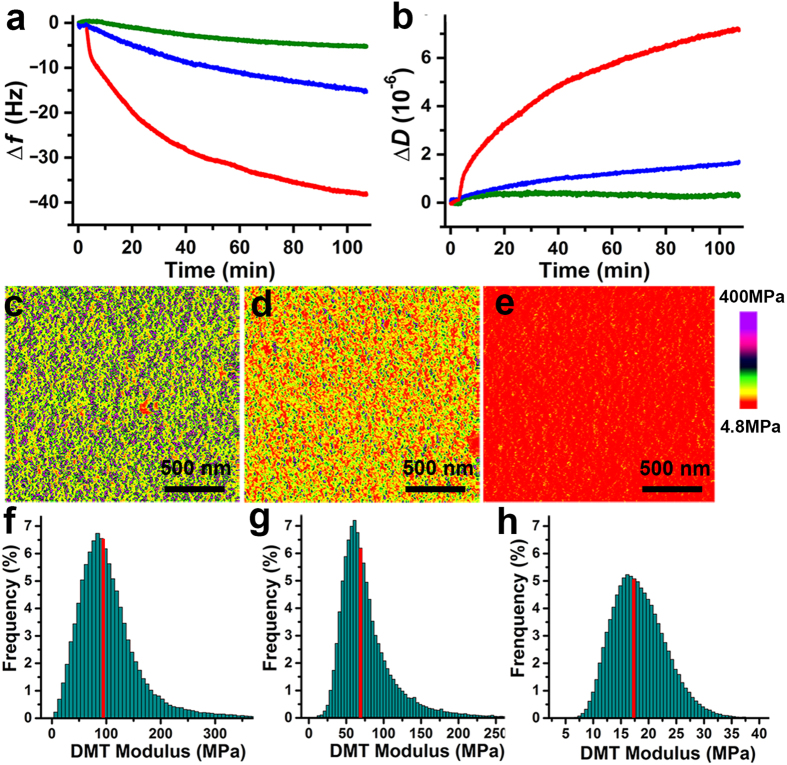
QCM and AMF measurements. Time dependence of frequency change (Δ*f*) (**a**) and dissipation change (Δ*D*) (**b**) of QCM resonators grafted with the PNI-*co*-PT-*co*-Glc copolymer film upon adsorption of FFF (red), LLL (blue) or EEE (green). Young’s modulus images (**c**–**e**) and moduli histograms (**f**–**h**) of the PNI-*co*-PT-*co*-Glc copolymer film on flat silicon substrate before (**c**,**f**) and after being treated by EEE (**d**,**g**) and FFF (**e**,**h**), respectively.

Quartz crystal microbalance (QCM) with dissipation monitoring was used to further study the adsorption behaviors of various tripeptides on resonator surface grafted with the PNI-*co*-PT-*co*-Glc copolymer film. Under the same concentration, FFF exhibited a strong adsorption, inducing a frequency change (Δ*f*) of the resonator of about 39 Hz (Fig. 4a), corresponding to an adsorption quality of 230.1 ng∙cm^−2^. However, LLL and EEE showed much weaker adsorption (adsorption qualities were 88.5 and 29.5 ng∙cm^−2^, respectively). Moreover, the dissipation curve can well describe the change in conformation and viscoelasticity of polymer film^39^. As shown in Fig. 4b, the dissipation (Δ*D*) increased to about 7.2 × 10^−6^ upon the injection of FFF solution. By comparison, the values were only about 1.8 × 10^−6^ and 2 × 10^−7^ for LLL and EEE, respectively. Thus we reasonably presume that the copolymer film becomes more swollen and viscous upon adsorption of aromatic tripeptide FFF than other tripeptides.

In addition, it has been widely acknowledged that atomic force microscope (AFM) enables quantitative measurement of surface mechanical properties (e.g., stiffness and deformation) for polymer materials at the nanoscale^40^,^41^,^42^. Here, we attempted to employ AFM in PeakForce QNM mode to study the PNI-*co*-PT-*co*-Glc copolymer film on flat silicon substrate upon treatment by tripeptides for more information. Figure 4c–e show the AFM Young’s modulus images, in which clear colour change from green (high modulus) to yellow or red (low modulus) indicates that the film became softer upon treatments by tripeptides, while more significant change was caused by FFF. And statistical analyses further show that the average modulus (mean ± s.d.) of the film originally was 97.7 ± 5.6 MPa (Fig. 4f), which decreased to 72.9 ± 4.2 MPa (Fig. 4g) and 17.5 ± 1.5 MPa (Fig. 4h) after being treated by EEE and FFF, respectively, implying the copolymer film surface become much softer after being treated by aromatic tripeptide FFF^43^,^44^. Therefore, these data clearly indicate that the adsorption of aromatic peptide FFF via CH-π interaction causes conformational change of copolymer from relative rigid contract conformation to soft swollen conformation, which results in subsequent dramatic transition in surface viscoelasticity and stiffness. Meanwhile, surface morphology and roughness for the PNI-*co*-PT-*co*-Glc copolymer film were also obtained from AMF measurements. By comparing these AFM images, section profiles and roughness values, a reduction in surface roughness was observed for the copolymer film after being treated with aromatic tripeptide FFF, while no obvious change for EEE was observed (see Supplementary Fig. S10 and Table S3 for more details). These data reveal the adsorption of FFF leads to a slight swelling of the copolymer film, resulting in the reduction of surface roughness, which contributes to surface wettability change to some extent^45^.

**Figure 5:**
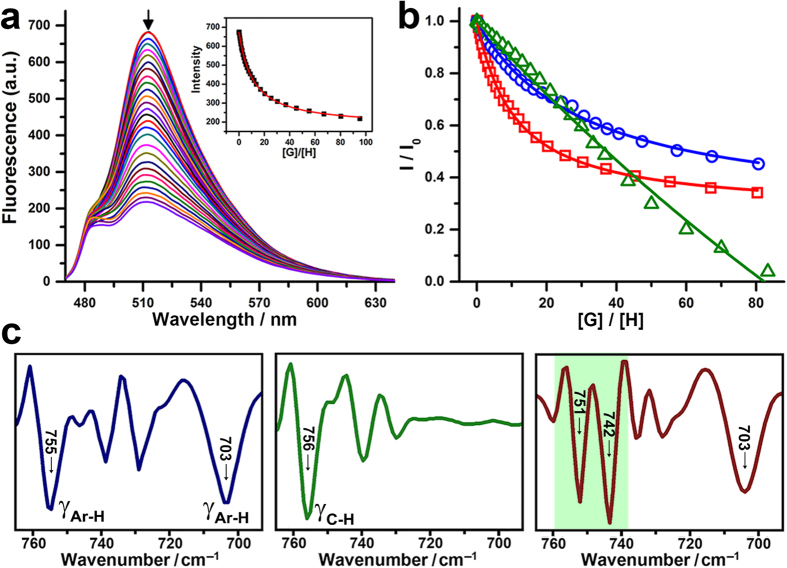
Investigation of CH-π interaction on single molecular level. (**a**) Fluorescence spectra of fluorescein-labeled Glc upon addition of various amounts of aromatic tripeptide FFF in Tris-buffer solution. (**b**) Fluorescent intensity changes of fluorescein-labeled Glc solution upon addition of various amounts of FFF (red), LLL (blue) and EEE (green), respectively. (**c**) Representative FT-IR second derivative spectra of FFF (blue), Glc (green) and mixture of FFF with equimolar amount of Glc (red) in DMSO-*d*_6_.

To further determine the CH-π interaction from molecule level, fluorescent titration experiments between saccharide units and tripeptides were performed. Results showed that the addition of FFF caused obvious fluorescence quenching for the fluorescein-labeled monosaccharide Glc (Fig. 5a), giving an association constant (*K*_a_) of 9.87 × 10^4^ L∙mol^−1^, which is almost 3 times and 25 times larger than those of LLL and EEE, respectively (see Supplementary Table S4). And the fitting curves of fluorescent intensity changes further confirmed this difference in *K*_a_ values (Fig. 5b). In addition, a similar tendency in *K*_a_ values was observed for fluorescein-labeled AcGlc (AcGlc denotes the tetra-acetylated Glc) upon addition of tripeptides (see Supplementary Table S4). Therefore, the above two set of binding data indicated that CH groups rather than OH groups in saccharide contributed to the complexation between saccharide unit and aromatic peptide. On the other hand, FT-IR spectroscopy in a Bio-ATR mode was also used to investigate the complexation in solution^46^. As shown in Fig. 5c, the peaks associated with the bending vibration of Ar-H in FFF (755 cm^−1^) and C-H in Glc (756 cm^−1^) exhibit obvious redshifts due to the shielding effect caused by complexation. These changes indicated that both aromatic groups and CH groups were involved in the complex formation via CH-π interaction. And the ^1^H NMR investigation further confirmed the participation of aromatic groups in complexation (see Supplementary Fig. S11)^25^.

**Figure 6:**
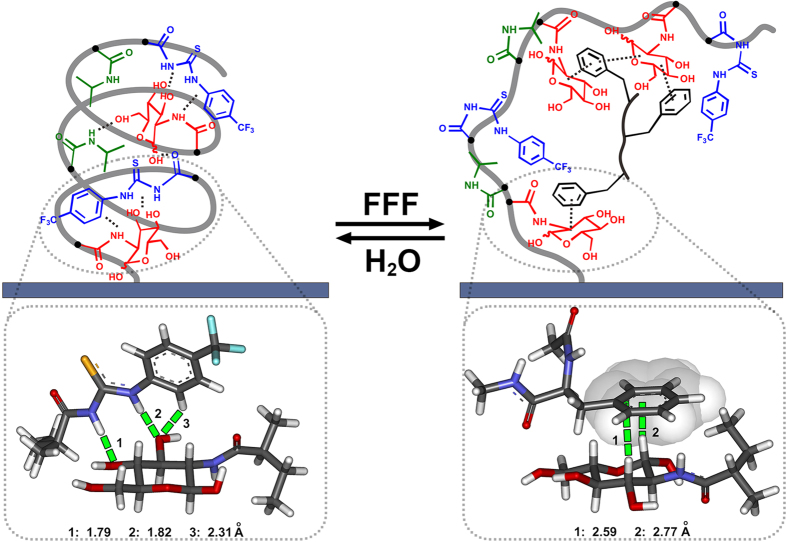
Potential contraction-swelling conformational transition process of the PNI-*co*-PT-*co*-Glc copolymer chains triggered by formation and dissociation of CH-π interaction. The lower parts show optimized interaction model of monosaccharide (Glc) with phenylthiourea (PT) (left), Glc with aromatic ring of FFF (right), calculated by quantum chemistry calculations at the single-molecule level (Gaussian, density functional theory at 6–311G level).

It has been proved that the incorporation of hydrophilic or hydrophobic groups into PNIPAAm chains would induce the variation of lower critical solution temperature^47^,^48^,^49^,^50^, and thus greatly influence the chain behaviors at a given temperature, due to the isothermal phase transition caused by the alteration of hydrophilic/hydrophobic balance of polymer chains^49^. In this system, initially, taking advantages of hydrogen bonding interactions between hydroxyls in Glc units and neighboring amides in PNIPAAm units or thiourea groups in PT units, such three components construct a hydrogen bonding network within copolymer chains (as depicted in Fig. 6)^51^, which results in a contracted conformation for the copolymer chains and a relatively hydrophobic property for the copolymer film. Moreover, the presence of CF_3_ groups in PT units may further enhance the initial surface hydrophobicity^45^. Then, when being immersed in tripeptide FFF solution, monosaccharide Glc units in copolymer chains combine the aromatic rings of FFF and form complexes via CH-π interaction (interaction model was shown in Fig. 6, bottom-right), which leads to the collapse of the initial hydrogen bonding network, and also breaks the initial amphiphilic balance of copolymer chains^52^. Consequently, copolymer chains stretched and become swollen, accompanied by the exposure of more hydrophilic moieties (e.g., imide groups, hydroxyl groups) embedded initially in contracted structure, which was reflected in hydrophilic surface with a smaller contact angle^53^,^54^,^55^,^56^. Therefore, a new amphiphilic balance for copolymer chains is reconstructed. Then, further treatment of pure water destroys of the complex among monosaccharides and FFF, and extrudes FFF into environmental water, thus the copolymer chain returns to its initial contracted state. This contraction-swelling conformational transition for copolymer film is in excellent accordance with the QCM and AFM experiments and dramatic switching in surface macroscopic properties on the film described above.

## Conclusion

In summary, we presented a novel CH-π interaction driven macroscopic property transition on a smart polymer surface with an R-M-F designing strategy. This new driving force is fundamentally important for the development of smart bioinerface materials, particularly those aiming at the applications for biology and biomedicine. For example, by taking advantages of the dramatic transition of surface wettability and adhesion, the dynamic adhesion and spreading process of protein or cell on materials surface might be controlled by small molecular peptides^57^,^58^, and this strategy with easy operation could be envisaged to be more suitable for bio-related applications^59^. On the other hand, this finding provides a new insight into disclosing the mysteries of CH-π interaction, the significance of which is underestimated in the previous research due to the ultra-weak affinity and the lack of efficient approach to amplify this effect to the macroscopic level. Further research will promote novel applications in the fields of peptides separation, bio-manipulation and bio-sensing, etc.

## Methods

### Materials

*N*-Isopropylacrylamide (NIPAAm, 99%, Acros) was purified by recrystallization in n-hexane for three times prior to use. The monosaccharide monomer (Glc), tetra-acetylated monosaccharide monomer (AcGlc), trifluoromethyl phenylthiourea monomer (PT), and various tripeptides were synthesized (see Supplementary information for details). Toluene, dichloromethane, methanol, and pyridine were distilled and dried before use according to standard procedures. Cu(I)Br (99.998%, Alfa Aesar), 3-aminopropyl-trimethoxysilane (ATMS, 97%, Sigma-Aldrich), α-bromoisobutyryl bromide (BiBB, 98%, Sigma-Aldrich), *N*,*N*,*N*′,*N*′,*N*′-pentamethyldiethylenetriamine (PMDETA, 99%, Sigma-Aldrich) were used as received. Double distilled water (18.2 MΩ∙cm, MilliQ system) was used, and other general solvents and chemicals were used as received.

### Preparation of the copolymer film

A clean silicon wafer (10 × 10 mm) was immersed in aqueous NaOH (0.1 mol∙L^−1^) for 8 minutes and subsequently in HNO_3_ (0.1 mol∙L^−1^) for 15 minutes to generate surface hydroxyl groups. After the wafer had been washed with an excess of water and dried under a flow of nitrogen gas, it was heated to reflux in toluene that contained ATMS (5%, w/v) for 6 hours to obtain chemically bonded –NH_2_ groups on the surface. The surface was rinsed with toluene and dichloromethane to remove remaining ATMS, dried under a flow of nitrogen gas, and immersed in dry 10 mL dichloromethane that contained 200 μL pyridine. The polymerization initiator BiBB (200 μL) was added dropwise into the as-prepared solvent containing the silicon wafer at 0 °C, the mixture was left for 1 hour at this temperature, and then at room temperature for additional 12 hours. The bromine-substituted silicon wafer was rinsed with dichloromethane for three times, and dried under a flow of nitrogen gas. Polymerization was achieved by immersing the chemically modified silicon wafer in a degassed solution of NIPAAm (0.792 g, 7 mmol), PT (0.41 g, 1.5 mmol) and Glc (0.35 g, 1.5 mmol) in a mixture of water (3 mL), methanol (3 mL) and DMF (6 mL) containing CuBr (0.032 g, 0.23 mmol) and PMDETA (0.16 mL) for 6 hours at 60 °C. Similar protocol was used to prepare the reference polymer film PNI-*co*-PT-*co*-AcGlc, PNI-*co*-PT, PNI-*co*-Glc, and PNIPAAm on flat silicon substrates. In addition, preparations of the copolymer film on textured silicon substrate and Au-coated QCM resonator were also used the same protocol described above (see Supplementary information for more details).

### Contact angle measurements

A series of tripeptide solutions with different concentrations ranging from 1 × 10^−5^ to 1 × 10^−2^ mol·L^−1^ in pure water were prepared in advance (maximum concentration for FFF or WWW is 3 × 10^−5^ mol·L^−1^). Before measurement, a silicon substrate grafted with the copolymer film was immersed in the tripeptide solution for 20 minutes, and then dried under a flow of nitrogen gas to move any remaining excess liquid. Then the silicon substrate with copolymer film was placed on the sample stage of DataPhysics OCA35 goniometer, and a water droplet of 3 μL was carefully deposited on the film with a precise electric dosing syringe. The static CA was recorded for each substrate using the sessile drop method at ambient atmosphere and a constant temperature of 25 °C. Each measurement was repeated several times to ensure the reliability of data. The dynamic CA (advancing CA and receding CA) was measured by sessile drop method, the outer diameter for the needle used was about 0.23 mm. The advancing CA (*θ*_adv_) and receding CA (*θ*_rec_) were measured during the expansion and contraction of a water droplet, respectively. The volume of the water droplet increased or decreased with a constant injection and withdrawal rate of 1 μL·s^−1^ in the range of 2–10 μL. The data were recorded synchronously when the edge of the water drop expanded or contracted constantly.

### Adhesion measurements

Surface water adhesive force was measured at ambient atmosphere and a constant temperature of 25 °C. Firstly, water droplet of 5 μL was suspended on the metal ring of high-sensitivity microelectromechanical balance system. The silicon substrate grafted with the PNI-*co*-PT-*co*-Glc copolymer film was placed on the lifting table. The lifting table moved upward at a constant speed of 0.1 mm·s^−1^ until the film contacted the water droplet. After the instantaneous contact, the substrate continued to move 0.1 mm closer toward the water droplet. Then, the substrate began to move downward at a constant speed of 0.1 mm·s^−1^. The values of adhesive force was recorded continuously. Each measurement was conducted at least three times in different position of a substrate.

### QCM measurements

Firstly, the copolymer-modified Au-coated QCM resonator was put into a flow-cell of Q-Sense E4 system. After stabilization of fundamental resonance frequency with the Phosphate buffer (PB) solution (NaH_2_PO_4_/Na_2_HPO_4_, 0.1 mmol·L^−1^, pH 7.4), tripeptide solution with a constant concentration of 0.5 mg·mL^−1^ in PB solution was pumped into flow-cell at a flow rate of 100 μL·min^−1^. The frequency change (Δ*f*) and dissipation change (Δ*D*) were recorded simultaneously. The adsorption quantities (A) were calculated according to the equation: A = Δ*f* × 17.7/n, ng·cm^−2^, where n is the overtone number.

### AFM measurements

The surface stiffness of copolymer film was investigated by AFM in PeakForce QNM mode. The system was calibrated by using the absolute method recommended by Bruker’s user manual before each experiment. On scan parameters, ScanAsyst Auto Control was set to ON, scan rate was set at 1 Hz. Firstly, the as-prepared the PNI-*co*-PT-*co*-Glc copolymer film sample was measured and obtained an original result. Then, the copolymer film was immersed in tripeptide solutions with an identical concentration of 3 mmol·L^−1^ for 20 minutes. After that the surface was dried under a flow of nitrogen gas to remove any remaining excess liquid, then AFM measurement for the copolymer film was performed.

## Additional Information

**How to cite this article**: Li, M. *et al.* CH-π Interaction Driven Macroscopic Property Transition on Smart Polymer Surface. *Sci. Rep.* **5**, 15742; doi: 10.1038/srep15742 (2015).

## Supplementary Material

Supplementary Information
