# Supplementary material for: CH-π Interaction Driven Macroscopic Property Transition on Smart Polymer Surface
Source: Sci Rep. 2015 Oct 29;5:15742. doi: 10.1038/srep15742 (PMC4625179; doi:10.1038/srep15742)
Supplement: Supplementary Information [file srep15742-s1.pdf]

Supplementary Information

CH-π Interaction Driven Macroscopic Property Transition on Smart Polymer Surface

Minmin Li[1], Guangyan Qing[1], Yuting Xiong[1], Yuekun Lai[3] & Taolei Sun[1,2,*]

[1]State Key Laboratory of Advanced Technology for Materials Synthesis and Processing, Wuhan University of Technology, 122 Luoshi Road, Wuhan 430070, P. R. China. [2]School of Chemistry, Chemical Engineering and Life Science, Wuhan University of Technology, 122 Luoshi Road, Wuhan 430070, P. R. China. [3]College of Textile and Clothing Engineering, Soochow University, 199 Ren'ai Road, Suzhou 215123, P. R. China

Correspondence and requests for materials should be addressed to T.S. (Email: suntl@whut.edu.cn).

1. Experimental materials and instruments

D-(+)-glucosamine hydrochloride (98%, TCI) and 4-aminobenzotrifluoride (99%, J&K), triisopropylsilane (TIS, 98%, Alfa Aesar), trifluoroacetic acid (TFA, 99%, Acros), triethylamine (TEA, 99%, Acros), 2-mercaptoethylamine hydrochloride (MEAH, 98%, Alfa Aesar) were used as received. Fmoc-Glu(OtBu)-OH, Fmoc-Val-OH, Fmoc-Ser-OH, Fmoc-Leu-OH, Fmoc-Phe-OH, Fmoc-Trp-OH, Fmoc-Asp(OtBu)-OH, Fmoc-Glu(OtBu)-Wang resin, Fmoc-Val-Wang resin, Fomc-Ser-Wang resin, Fmoc-Leu-Wang resin, Fmoc-Phe-Wang resin, Fmoc-Trp-Wang resin, Fmoc-Asp(OtBu)-Wang resin, O-(Benzotriazol-1-yl)-*N*,*N*,*N*',*N*'-tetramethyluronium hexafluorophosphate (HBTU, 99%), *N*,*N*-diisopropylethylamine (DIEA, 98%) were purchased from GL BioChem. Ltd. (Shanghai, China) and used as received.

The synthesis of various tripeptides was achieved by using CS-Bio Peptide Synthesizer CS 136XT (USA) and purified through Shimadzu UFLC 20A purity system with a C18 reversed-phase high-performance chromatographic column (HPLC, particle size: 5 μm, 4.6 × 250 mm and 10 × 250 mm, Inertsil ODS-SP). NMR spectra were recorded on a Bruck AVANCE III 500-MHz spectrometer. Mass spectra were obtained with a Finnigan LCQ advantage mass spectrometer. Fluorescence spectra were recorded on a PerkinElmer LS55 fluorescence spectrophotometer. X-ray photoelectron spectroscopy (XPS) was obtained with a VG Multilab 2000. Atomic force microscopy (AFM) investigations were conducted by Bruker Multimode 8 AFM with the ScanAsyst mode and quantitative nanomechanical mapping (QNM) mode. FI-IR spectra were recorded with a Bruker Vertex 80V FT-IR spectrometer in an ATR mode and a Bio-ATR mode. Elemental analysis was determined with a Carlo-Erba 1106 instrument. Scanning electron microscopy (SEM) spectra were recorded on a Hitachi S-4800 SEM. Static and dynamic water contact angle (CA) measurements were conducted using the sessile drop method on a DataPhysics OCA35 goniometer with software SCA20. The water adhesive forces were measured with a commercial tensiometer high-sensitivity micro-electro-mechanical balance DataPhysics DCAT 11 with software SCAT 37. Dynamic adsorption experiments were conducted on a quartz crystal microbalance with dissipation monitoring (QCM-D) (Q-Sense E4 System, Biolin Scientific Corp. Sweden).

2. Synthesis and additional preparation procedures

2.1 Synthesis of *N*-acryloyl-D-glucosamine monomer (Glc):

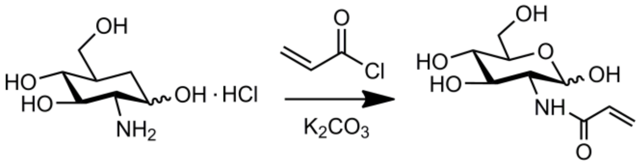

D-(+)-glucosamine hydrochloride (5.0 g, 23.2 mmol) was dissolved in 50 mL 2 mol L^{-1} K_2CO_3 aqueous solution. The reaction vessel was subsequently cooled to 0 ℃, and then acryloyl chloride (2.5 mL, 28 mmol) was added dropwise into the reaction solution under vigorous stirring. The mixture solution was maintained at 0 ℃ for 2 hours and continued to react for 12 hours at room temperature. Then, the reaction solution was washed with diethyl ether for three times. After the evaporation of water of the reaction solution, the solid crude product was further dissolved in methyl alcohol and purified by silica gel column with an eluent of methanol/ethyl acetate (20/80, v/v). The pure product was obtained by recrystallization twice in a methanol/ethyl acetate mixture solvent (20/80, v/v) at 4 ℃ (1.30 g, yield: 24%, m.p. 130 ~ 135 ℃). ^{1}H NMR (500 MHz, D_2O): δ (ppm): 6.46-6.17 (m, CO-C*H*, CH=C*H*, anti), 5.98-5.71 (CH=C*H*, syn), 5.28 (d, *J* = 3.5 Hz, C*H*), 4.13-3.43 (C*H* × 5). ^{13}C NMR (500 MHz, DMSO-d_6): δ (ppm): 165.12, 132.59, 125.25, 90.94, 72.53, 71.55, 70.78, 61.54, 54.97. IR spectrum (3423, 3325, 3275, 2924, 1652, 1609, 1550, 1442, 1410, 1351, 1330, 1285, 1266, 1238, 1144, 1119, 1101, 1054, 1037, 1018, 990, 955, 863, 846, 806, 785, 740 cm^{-1}). MADLI MS: m/z calcd. for $C_9H_{15}NO_6$: 233.09; found: 256.03 $[M+Na]^+$. Elemental analysis calcd. for $C_9H_{15}NO_6$ (%): C, 46.35; H, 6.48; N, 6.01. Found: C, 45.86; H, 6.93; N, 5.89.

2.2 Synthesis of *N*-acryloyl-1,3,4,6-tetraacetyl-D-glucosamine monomer:

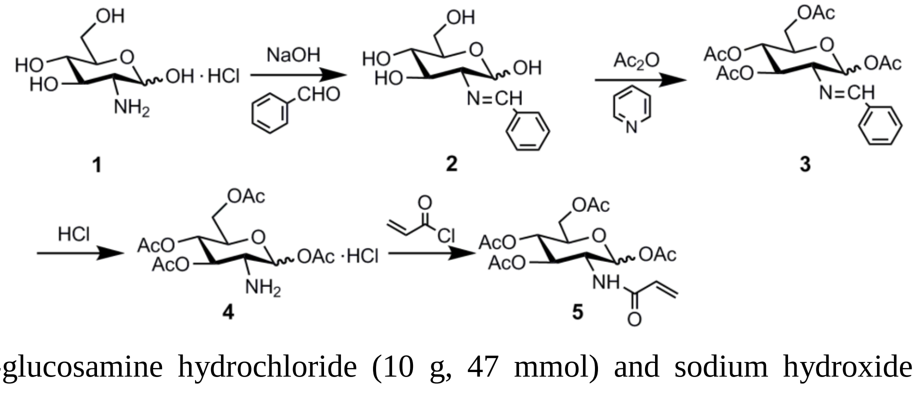

D-(+)-glucosamine hydrochloride (10 g, 47 mmol) and sodium hydroxide (2.4 g, 60 mmol) were dissolved in 47 mL water, the solution was stirred at −10 ℃ for 10 minutes. Under vigorous stirring, 4.9 mL benzaldehyde was added dropwise into the solution.

Subsequently, a large amount of white precipitate appeared gradually. Then the product **2** was obtained by filtering and washing with cold water and mixture of ethanol and diethyl ether (1/1, v/v) (9.82 g, yield: 78%, m.p. 180 ~ 185 ℃)[1].

Reactant **2** (8.01 g, 30 mmol) was dissolved in 50 mL dry pyridine, and the solution was cooled to −10 ℃. Under vigorous stirring, 25 mL of acetic anhydride was added dropwise into the solution, and the stirring was continued for 1 hour at −10 °C. Subsequently, the reaction was slowly warmed to room temperature and continued to react for 18 hours. Then the reaction solution was poured into cold water and mixed strongly. The product **3** was obtained by filtering and washing with cold water and petroleum ether (11.2 g, yield: 86%, m.p. 156 ~ 160 ℃)[1].

Reactant **3** (10 g, 23 mmol) was dissolved in 100 mL ethyl acetate and the solution was heated to reflux. Under vigorous stirring, 1.8 mL of hydrochloric acid (37%) was added dropwise into the mixture solution, and then white precipitate appeared gradually. The product **4** was obtained after filtration and drying under vacuum (7.12 g, yield: 80%, m.p. 230 ~ 235 ℃)[1].

Reactant **4** (5.0 g, 13.1 mmol) and TEA (0.69 mL, 5 mmol) was added into 40 mL dry dichloromethane, the mixture solution was cooled to 0 ℃ and stirred for 10 minutes. Then acryloyl chloride (1.26 mL, 15 mmol) was added dropwise into the solution, and continued to stir at 0 ℃ for 5 hours. Subsequently, the reaction solution was washed with sodium bicarbonate solution (5%, wt%) and cold water for three times respectively, and then the organic layer was collected and dried with anhydrous Na_2SO_4 overnight. After filtration and evaporation of solvent, the target product **5** was obtained as white solid (3.58 g, yield: 68%, m.p. 174 ~ 180 ℃). ^{1}H NMR (500 MHz, DMSO-d_6): δ (ppm): 8.23 (d, J = 9.2 Hz, 1H, CON*H*), 6.17 − 6.03 (m, 2H, C=C*H*), 5.78 (d, J = 8.8 Hz, 1H, C−C*H*), 5.62 (d, d, J_1 = J_2 = 3.0 Hz, 1H, C−C*H*), 5.23 (t, J_1 = J_2 = 10.0 Hz, 1H, C−C*H*), 4.92 (t, J_1 = J_2 = 9.6 Hz, 1H, C−C*H*), 4.19 (d, d, J_1 = J_2 = 4.5 Hz, 1H, C−C*H*), 4.09 − 3.95 (m, 3H, C−C*H*), 2.02 (d, J = 4.2 Hz, 6H, C−CH_3), 1.98 (s, 3H, C−CH_3), 1.89 (s, 3H, C−CH_3). ^{13}C NMR (500 MHz, DMSO-d_6): δ (ppm): 170.49, 169.98, 169.72, 169.28, 165.33, 131.60, 126.64, 92.17, 72.68, 72.03, 68.51, 61.95, 52.53, 20.96, 20.86, 20.73. IR spectrum (3310, 2957, 1745, 1665, 1635, 1611, 1537, 1404, 1369, 1323, 1215, 1113, 1075, 1032, 976, 908, 803, 701 cm^{-1}). MADLI MS: m/z calcd. for $C_{17}H_{23}NO_{10}$: 401.13; found: 424.12 $[M+Na]^+$. Elemental analysis calcd. for $C_{17}H_{23}NO_{10}$ (%): C, 50.87; H, 5.78; N, 3.49. Found: C, 50.80; H, 5.83; N, 3.51.

2.3 Synthesis of *N*-acryloyl-*N′*-[4-(trifluoromethyl)-phenylthiourea monomer:

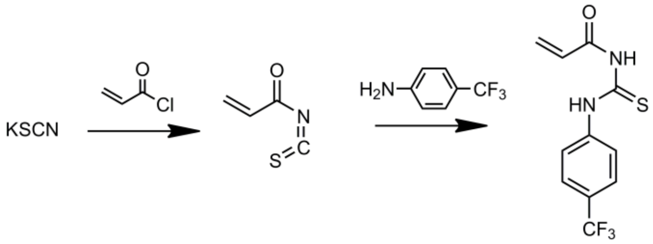

Acryloyl chloride (1.21 mL, 15 mmol) was added dropwise into a solution of KSCN (1.45 g, 15 mmol) in 30 mL dry acetone, and the reaction solution was stirred at ambient temperature overnight. After filtration, the yellow filtrate was collected and stored in a flask for direct use in the next step. 4-Aminobenzotrifluoride (1.88 mL, 15 mmol) was dissolved in 30 mL dichloromethane and stirred for 10 minutes. Then the yellow filtrate obtained above was added dropwise into the mixture solution with 4-aminobenzotrifluoride, followed by stirring for 12 hours. Then, the reaction solution was washed with water for three times, and the organic phase was dried with anhydrous Na_2SO_4. After filtration and concentration, yellow needle-shaped crystals were obtained. Then, these crude crystals were recrystallized in dichloromethane twice to give the pure product (1.4 g, yield: 35%, m.p. 115 ~ 120 ℃). ^1H NMR (500 MHz, DMSO-d_6): δ (ppm): 12.72 (s, 1H, CO−N*H*), 11.74 (s, 1H, CO−N*H*), 7.91 (d, J = 8.4 Hz, 2H, Ph−*H*), 7.74 (d, J = 8.5 Hz, 2H, Ph−*H*), 6.61 (d, d, $J_1 = J_2$ = 10.2 Hz, 1H, C=C*H*), 6.44 (d, d, $J_1 = J_2$ = 17.1 Hz, 1H, C=C*H*), 6.00 (d, d, $J_1 = J_2$ = 9.9 Hz, 1H, C=C*H*). ^{13}C NMR (500 MHz, DMSO-d_6): δ (ppm): 179.72, 166.10, 141.91, 132.51, 130.21, 126.29, 126.26, 124.94. IR spectrum (3148, 3023, 1940, 1690, 1630, 1613, 1594, 1527, 1405, 1314, 1246, 1168, 1109, 1062, 1016, 977, 894, 851, 799, 741, 727, 682 cm^{-1}). MADLI MS: m/z calcd. for $C_{11}H_9F_3N_2OS$: 274.04; Found: 275.07 $[M+H]^+$. Elemental Analysis calcd. for $C_{11}H_9F_3N_2OS$ (%): C, 48.17; H, 3.31; N, 10.21; S, 11.69. Found: C, 48.01; H, 3.25; N, 10.23; S, 11.79.

2.4 Synthesis and characterization of various tripeptides:

A standard fluorenylmethoxycarbonyl (Fmoc)-based solid-phase peptide synthesis method was adopted to automatically synthesize various tripeptides used in this work by a peptide synthesizer. Specifically, tripeptides were synthesized starting from Wang resin. The coupling reagents and amino acids were dissolved in *N*,*N*-dimethyl formamide (DMF) with desired concentrations, respectively. Each coupling reaction containing Fmoc-amino acid (4 equiv.), DIEA (8 equiv.) and HBTU (4 equiv.) was allowed to perform for 90 minutes. Each Fmoc-deprotection reaction was performed using 20% (v/v) piperidine in DMF for 30

minutes. After the final synthesis, the peptidyl-resin was washed for three times with dichloromethane and diethyl ether respectively, and dried under vacuum. Then, the peptide was cleaved from the Wang resin in TFA/TIS/water (95/2.5/2.5, v/v/v) solution for 4 hours. The crude product was precipitated by ice-cold diethyl ether and washed with diethyl ether for three times. Then the peptide was purified by a semi-preparative HPLC on C18 column (particle size: 5 μm, 10 × 250 mm) with a linear gradient of the binary solvent system of 0.1% TFA in water (solvent A) and 0.1% TFA in acetonitrile (solvent B) and UV detection at 220 nm. The purity was determined by HPLC (particle size: 5 μm, 4.6 × 250 mm, Inertsil ODS-SP C18 column). The purity and MS identification data of various tripeptides were listed as follows:

Phe-Phe-Phe (FFF): purity: 96.2%, HPLC separation parameters: a linear gradient of 10 − 90% B over 20 minutes at a flow rate of 1 mL min^{-1}. MADLI MS: m/z calcd. for $C_{27}H_{29}N_3O_4$: 459.22; found: 460.18 $[M+H]^+$.

Glu-Glu-Glu (EEE): purity: 93.4%, HPLC separation parameters: a linear gradient of 10 − 50% B over 10 minutes at a flow rate of 1 mL min^{-1}. MADLI MS: m/z calcd. for $C_{15}H_{23}N_3O_{10}$: 405.14; found: 406.16 $[M+H]^+$.

Ser-Ser-Ser (SSS): purity: 94.7%, HPLC separation parameters: a linear gradient of 2 − 50% B over 8 minutes at a flow rate of 1 mL min^{-1}. MADLI MS: m/z calcd. for $C_9H_{17}N_3O_7$: 279.11; found: 280.12 $[M+H]^+$.

Leu-Leu-Leu (LLL): purity: 95.5%, HPLC separation parameters: a linear gradient of 10 − 80% B over 15 minutes at a flow rate of 1 mL min^{-1}. MADLI MS: m/z calcd. for $C_{21}H_{41}N_3O_4$: 357.26; found: 358.27 $[M+H]^+$.

Val-Val-Val (VVV): purity: 95.8%, HPLC separation parameters: a linear gradient of 10 − 80% B over 15 minutes at a flow rate of 1 mL min^{-1}. MADLI MS: m/z calcd. for $C_{21}H_{41}N_3O_4$: 315.22; found: 316.22 $[M+H]^+$.

Trp-Trp-Trp (WWW): purity: 96.5%, HPLC separation parameters: a linear gradient of 10 − 95% B over 20 minutes at a flow rate of 1 mL min^{-1}. MADLI MS: m/z calcd. for $C_{33}H_{32}N_6O_4$: 576.25; found: 577.26 $[M+H]^+$.

Asp-Asp-Asp (DDD): purity: 94.1%, HPLC separation parameters: a linear gradient of 10 − 40% B over 6 minutes at a flow rate of 1 mL min^{-1}. MADLI MS: m/z calcd. for $C_{12}H_{17}N_3O_{10}$: 363.09; found: 386.01 $[M+Na]^+$.

Phe-Phe (FF): purity: 95.5%, HPLC separation parameters: a linear gradient of 10 − 90% B over 18 minutes at a flow rate of 1 mL min^{-1}. MADLI MS: m/z calcd. for $C_{27}H_{29}N_3O_4$: 312.15; found: 313.16 $[M+H]^+$.

Phe-Phe-Phe-Phe (FFFF): purity: 95.0%, HPLC separation parameters: a linear gradient of 10 − 90% B over 18 minutes at a flow rate of 1 mL min^{-1}. MADLI MS: m/z calcd. for $C_{36}H_{38}N_4O_5$: 606.28; found: 607.29 $[M+H]^+$.

2.5 Preparation of textured silicon substrate

The textured silicon substrate with regular pillars array was obtained by lithography etching[2]. The dimension of the pillars was 10 × 10 μm, the height was 10 μm and the interval distance between each pillar was 15 μm, as shown in Fig. S4 (A, B). Then the nanofibrous structures on the top of pillars were further prepared by an aqueous chemical etching method. The silicon substrate with regular pillars array was carefully immersed in a solution of hydrofluoric acid with silver nitrate (the concentration of HF/$AgNO_3$ was 4.6 mol L^{-1} and 0.1 mol L^{-1} respectively) for 3 minutes at 50 ℃. After etching, the substrate was immersed in HNO_3 (20%) for 20 minutes to remove silver film, resulting in the nanofibrous structure on the top of the pillars, as shown in Fig. S4 (C, D).

2.6 Preparation of the copolymer film on an Au-coated QCM resonator:

A QCM resonator was firstly cleaned by a fresh mixture solution of water, ammonium hydroxide and H_2O_2 (5/1/1, v/v/v) for 10 minutes at 70 ℃. After being rinsed by double distilled water sufficiently and dried under a flow of nitrogen gas, the resonator was immersed in a solution of ethanol (10.0 mL) containing MEAH (22.7 mg) and TEA (22.3 mg) for 24 hours, to obtain amine-modified surface. Then the resonator was rinsed with ethanol and dichloromethane sufficiently, and dried under a flow of nitrogen gas. Subsequently, the amine-modified resonator was immersed in a mixture solution of dichloromethane (10 mL) and pyridine (0.1 mL). BIBB (0.1 mL) was added dropwise into this solution at 0 ℃, and the mixture solution was left for 1 hour at this temperature, and then at room temperature for additional 12 hours. After that, the bromine-substituted resonator was obtained and cleaned with dichloromethane sufficiently. The subsequent fabrication of the copolymer film on QCM resonator was achieved by the same protocol used for the flat silicon substrate.

3. HPLC experiments

The relative hydrophobicity scales of these tripeptides can be evaluated accurately by HPLC with different retention time using a hydrophobic column and a hydrophilic column, respectively. Each tripeptide solution was dissolved in methyl alcohol with an appropriate concentration, and then was filtered through a 0.20 μm nylon filter. Subsequently, each solution was taken individually for HPLC analysis and their retention time was recorded. To

evaluate the relative hydrophobicity scales, an Inertsil ODS-SP analytical column (particle size: 5 μm, 4.6 × 250 mm) was used for the analysis with a mobile phase of acetonitrile/water (20/80, v/v) and a flow rate of 1 mL min^{-1}, the wavelength of UV detection was set as 220 nm and the column temperature was 30 ℃. An Acchrom S series "Click Cys" analytical column (particle size: 5 μm, 4.6 × 250 mm) was used to evaluate the relative hydrophilicity scales of three tripeptides, and water was used as mobile phase.

4. AFM experiments

The general characterizations (film thickness and morphology) of the copolymer film were performed using AFM in the ScanAsyst mode with a Nanoscope V controller and the software Nanoscope v8.12. The resulting images were processed using Nanoscope Analysis v1.40.

The surface modulus of copolymer film was investigated by AFM in PeakForce QNM mode at ambient atmosphere and a constant temperature of 25 ℃.

In the PeakForce QNM mode, the reduced modulus E^* is obtained by fitting the retraction curve using the Derjaguin-Muller-Toporov (DMT) model[3]:

$$F = \frac{4}{3} E^* \sqrt{R(d - d_0)^3} + F_{Adh} \quad (1)$$

Where F is the force on the tip, F_{Adh} is the adhesion force, R is the tip end radius, d-d_0 is the distance between tip and sample. As a polymer brush film, we used 0.5 as the sample's Poisson ratio as recommended by Bruker's users guide for a sample with stiffness lower than 100 MPa. Since the sample's Poisson ratio is known, the Young's modulus of the sample (E_s) can been calculated by the following equation:

$$E^* = \left[\frac{1 - \nu_{tip}^{\ 2}}{E_{tip}} + \frac{1 - \nu_s^{\ 2}}{E_s} \right]^{-1} \quad (2)$$

Where ν_{tip} and E_{tip} are the Poisson's ratio and Young's modulus of the tip, ν_{s} and E_{s} are the Poisson's ratio and Young's modulus of the sample. We assumed that the tip modulus E_{tip} is infinite, and calculate the sample Young's modulus using the sample Poisson's ratio.

In this work, a standard SNL-10 A$^{\#}$ probe was used and calibrated by using the absolute method (recommended by Bruker's PeakForce QNM users guide, 004-1036-000) before each experiment. The deflection sensitivity (67.51 nm/V) was measured on a clean silicon wafer surface, the spring constant was 0.51 N/m by using thermal tuning method, the tip end radius (3.8 nm) was determined by scanning a TipCheck sample.

5. Fluorescence titration experiments

Host fluorescein-labeled saccharide Glc and its acetylated derivative AcGlc were prepared in Tris-buffer solution (1.0 mM, pH 7.4) for 5.0×10^{-4} mol L^{-1}, respectively. Various guest tripeptides were prepared to 0.01 and 0.001 mol L^{-1} of stock solution in H_2O. The work solutions were prepared by adding different volumes of guest solution to a series of test tubes, and then same amount of stock solution of host was added into each test tube, followed by dilution to 3 mL with Tris-buffer solution. After being shaken for 1 minute, the work solutions were measured immediately at 25 ℃. The association constant (K_a) values were obtained from fluorescence titration experiments according to intensity changes in the emission-peak maximum. Detailed K_a values are shown in Table S2. All error values were obtained by the results of nonlinear curve fitting, the correlation coefficient (R) of nonlinear curve fitting was over 0.99. The nonlinear calculation equation is listed as follow:

$$F = F_0 + \frac{F_{\lim} - F_0}{2C_0}\left\{C_H + C_G + \frac{1}{K_a} - \left[\left(C_H + C_G + \frac{1}{K_a}\right)^2 - 4C_H C_G\right]^{1/2}\right\} \qquad (3)$$

Where F represents the fluorescent intensity, and C_H and C_G are the corresponding concentrations of saccharide hosts and peptide guests, respectively.

6. NMR measurements

NMR experiments were performed to obtain the interaction details of saccharide monomer Glc or its acetylated derivative AcGlc with tripeptide FFF. Herein, DMSO-d_6 was chosen as the solvent because both saccharides and FFF were well soluble in it.

7. FT-IR measurements

FT-IR spectra were performed on a Bruker Vertex 80V FT-IR spectrometer in a Bio-ATR mode. The spectra were recorded in the range of 4000 − 650 cm^{-1} with a spectral resolution of 4 cm^{-1} and a scan number of 120. The samples were dissolved in DMSO-d_6 with identical concentrations of 10 mmol L^{-1}.

8. Supplementary Figures and Tables

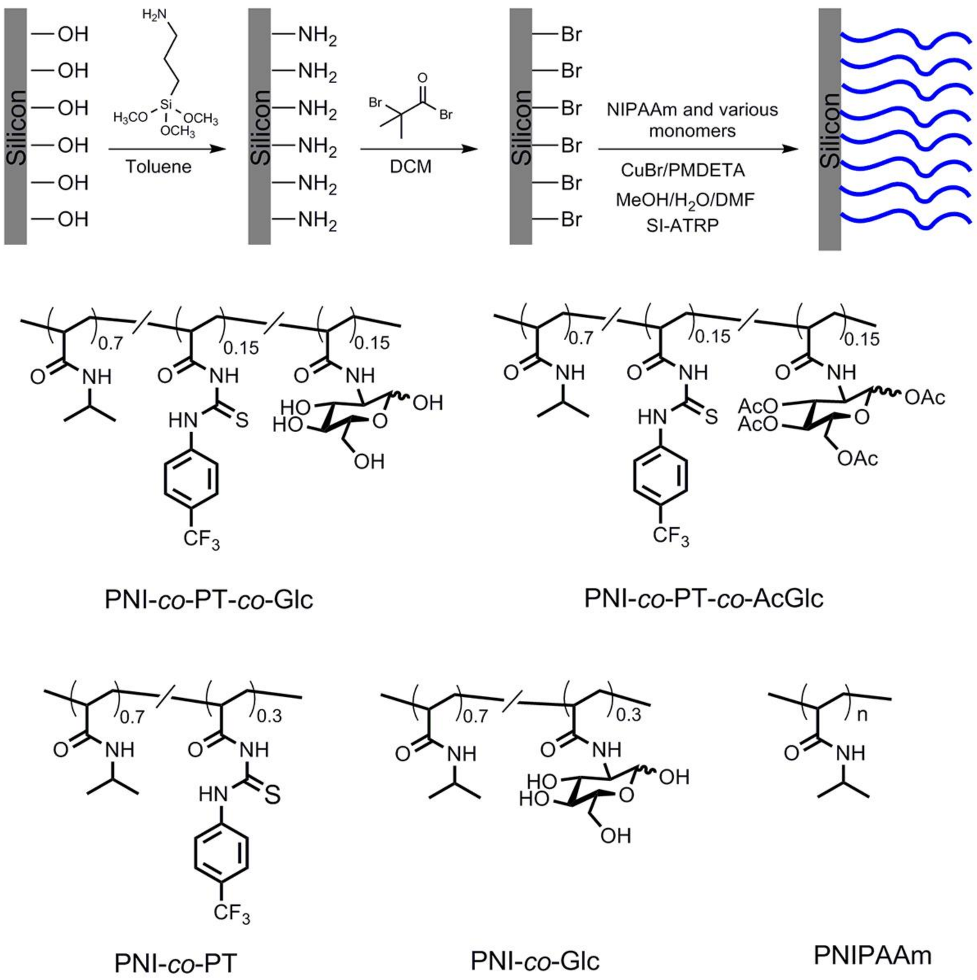

Figure S1. Functional copolymer was grafted onto a flat silicon substrate by surface initiated atom transfer radical polymerization (SI-ATRP). Schematic procedure for the preparation of polymer films on silicon substrate by SI-ATRP and the molecular structures of various polymers: PNI-*co*-PT-*co*-Glc, PNI-*co*-PT-*co*-AcGlc, PNI-*co*-PT, PNI-*co*-Glc, PNIPAAm.

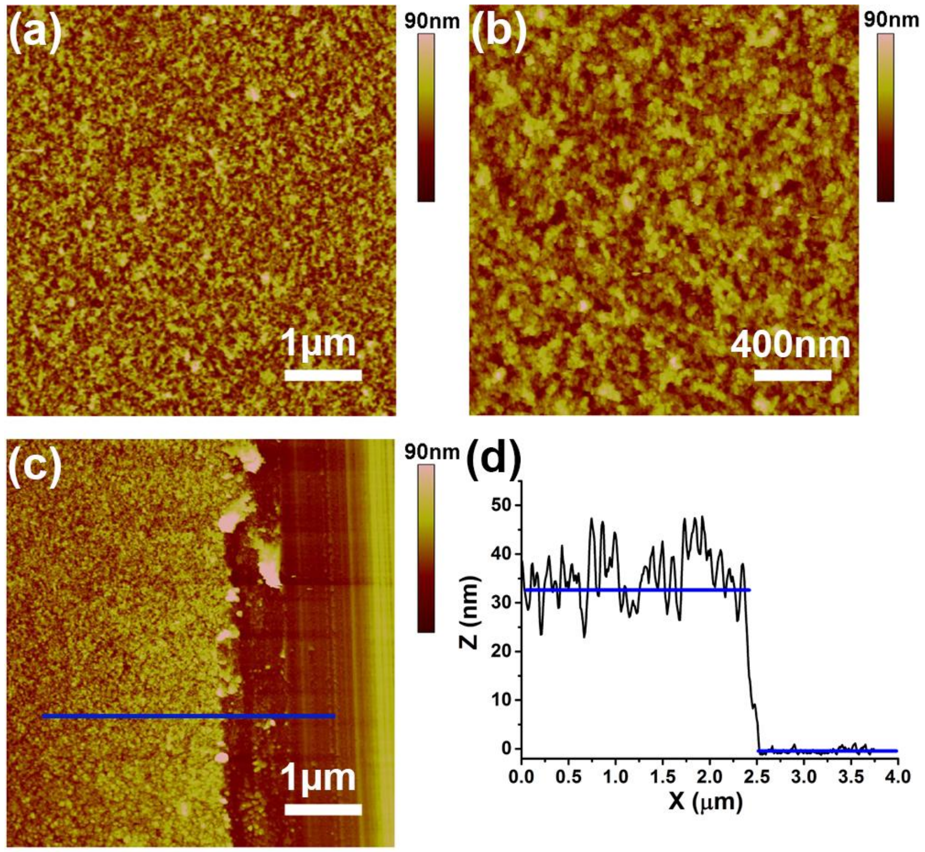

Figure S2. AFM images of copolymer film. (a, b) Large scale (a) and magnified (b) AFM height images of the PNI-*co*-PT-*co*-Glc copolymer film on flat silicon substrates. (c, d) Section profile (d) for the corresponding AFM image (c) along the blue line. The thickness of the copolymer film is about 33 ±5 nm.

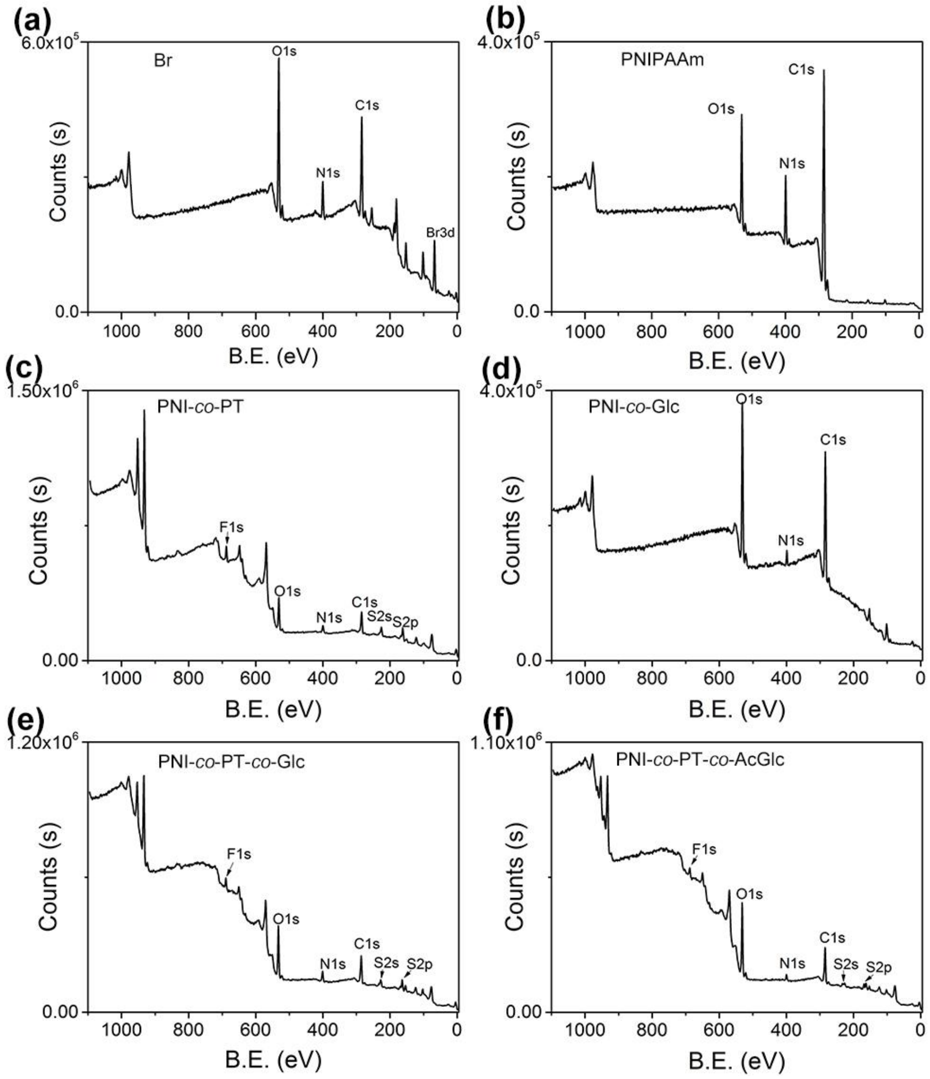

Figure S3. **XPS spectra of various film surface.** XPS spectra of Br-substituted monolayer (a), PNIPAAm (b), PNI-*co*-PT (c), PNI-*co*-Glc (d), PNI-*co*-PT-*co*-Glc (e) and PNI-*co*-PT-*co*-AcGlc (f) film on flat silicon substrates. In this experiment, XPS was employed to measure the elemental compositions of various films. The appearance of Br_{3d} peak clearly indicated that the initiator BiBB had been successfully immobilized on the silicon surface (Fig. S2a). Moreover, the elemental composition of the polymer film surface calculated from the XPS data was similar with the theoretically calculated composition of this polymeric film, as illustrated in Table S1. Combined with the appearance of the characteristic peaks of sulphur and fluorine, these data clearly demonstrated that various functional polymers with different components had been successfully grafted onto the flat silicon substrate.

Table S1. Elemental compositions of various polymer films calculated from XPS data.

Film surface		C %	N %	O %	S %	F %
PNIPAAm	Measured	68.84	13.74	13.86	0	0
	Calculated	63.68	12.38	14.14	0	0
PNI-*co*-PT	Measured	56.47	10.7	10.06	5.48	9.24
	Calculated	55.88	11.22	9.86	5.93	10.54
PNI-*co*-Glc	Measured	60.85	15.46	4.98	0	0
	Calculated	55.5.	26.79	9.38	0	0
PNI-*co*-PT-*co*-Glc	Measured	58.16	8.88	16.81	4.62	4.37
	Calculated	56.17	10.18	17.69	3.04	5.40
PNI-*co*-PT-*co*-AcGlc	Measured	60.28	7.59	18.83	3.75	3.54
	Calculated	56.30	8.78	20.49	2.62	4.66

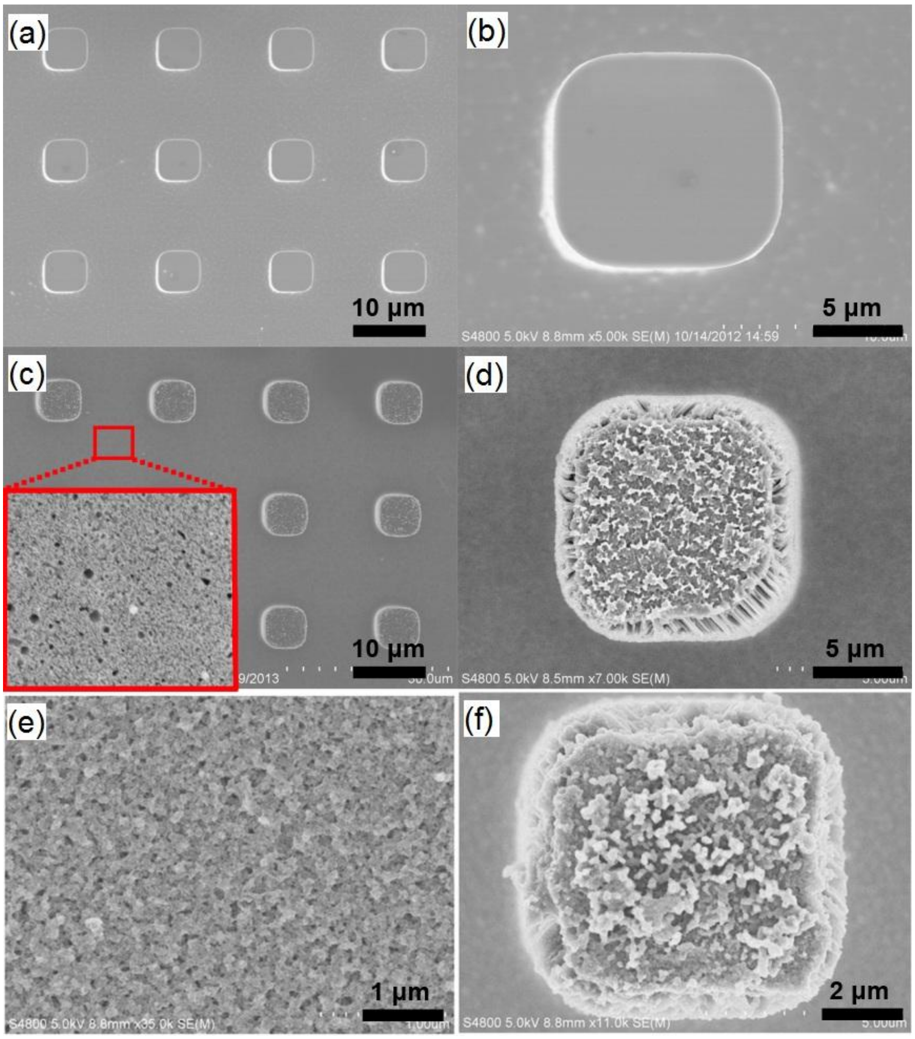

Figure S4. SEM images of textured silicon substrate before and after the modification of the PNI-*co*-PT-*co*-Glc copolymer film on it. (a, b) Large scale (a) and magnified (b) SEM images of regular pillars array of textured silicon substrate. (c, d) Large scale (c) and magnified (d) SEM images of nanofibrous structure of textured silicon substrate. The insert in Fig. S4c shows the magnified image of nanofibrous structure on the bottom of regular pillars array. (e, f) Morphological images of the bottom (e) and the top (f) of pillar after the modification of the PNI-*co*-PT-*co*-Glc copolymer film on the textured silicon substrate. These data indicated that the copolymer had been successfully grafted onto the textured silicon substrate.

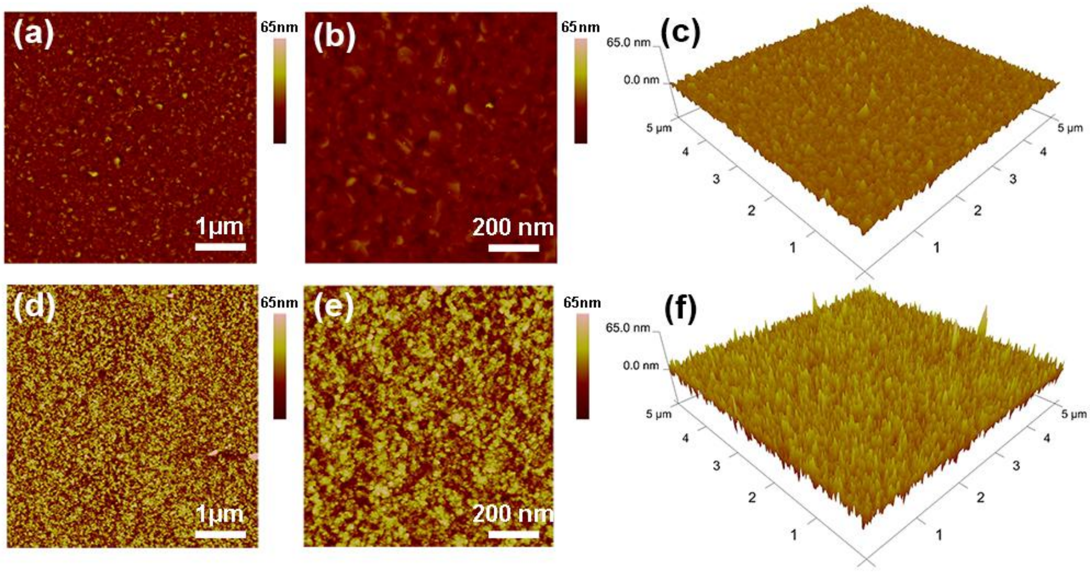

Figure S5. AFM height images of Au-coated QCM resonator before and after the modification of the PNI-*co*-PT-*co*-Glc copolymer film on it. (a-c) Large scale (a) and magnified (b) AFM images of Au-coated QCM resonator, and the corresponding three-dimensional height image (c). (d-f) Large scale (d) and magnified (e) AFM images of Au-coated QCM resonator modified with the PNI-*co*-PT-*co*-Glc copolymer film, and the corresponding three-dimensional height image (f). These images show that the resonator surface becomes much rough after the polymerization, accompanied by an obvious increase from 2.64 ± 1.43 to 10.32 ± 1.54 nm in mean roughness, which indicates the copolymer film has been successfully grafted onto the Au-coated QCM resonator surface.

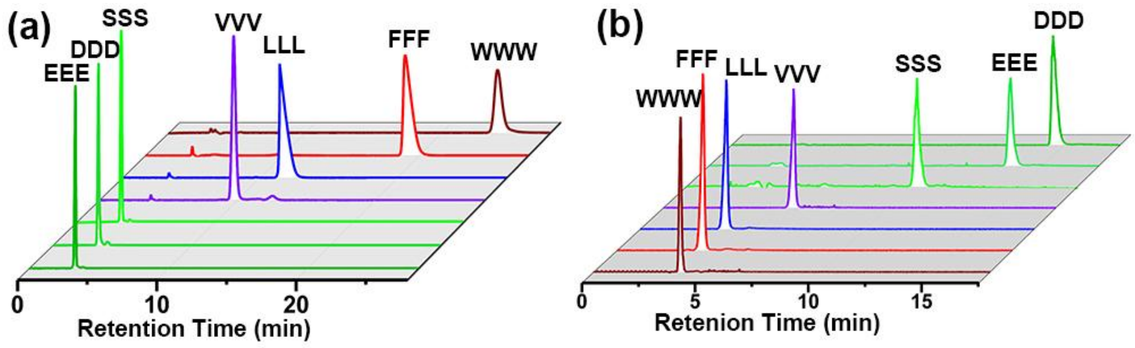

Figure S6. Retention time of tripeptides in HPLC. Relative hydrophobicity scales of various tripeptides evaluated by HPLC in a hydrophobic mode (a) and a hydrophilic mode (b). According to the typically chromatographic theory[4], different retention time represented the different hydrophobic or hydrophilic property of analytes under the same conditions (mobile phase: acetonitrile/water (20/80, v/v) used in the hydrophobic mode, pure water used in the hydrophilic mode; flow rate: 1 mL min^{-1}, UV detection (λ) : 220 nm, column temperature: 30 ℃). In hydrophobic mode (C18 as the stationary phase), EEE, DDD and SSS flowed along with the eluent, indicating that all of which were highly hydrophilic peptides. LLL and VVV with alkyl side chain represented the peptides with moderate hydrophilicity. However, the retention time of FFF and WWW was longer than other tripeptides, implying that FFF and WWW were relative hydrophobic peptides. However, On the other hand, the relative retention order of tripeptides in the hydrophilic mode (Click-Cys as the stationary phase) was completely contrary to that in a hydrophilic mode. Therefore, combined the results from this two HPLC modes, the relative hydrophobic properties of these model tripeptides was WWW>FFF>LLL>VVV>SSS>EEE>DDD.

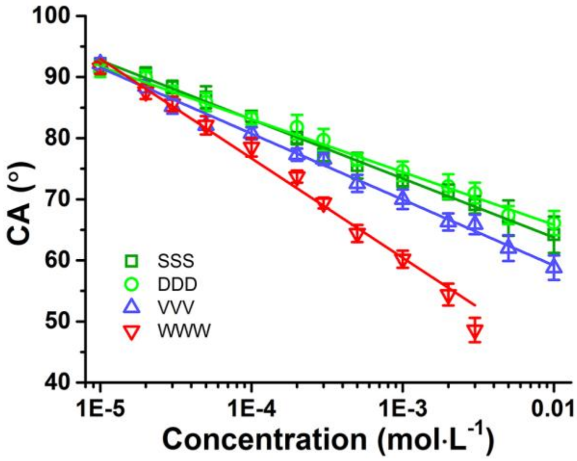

Figure S7. Relationship between CAs of the PNI-*co*-PT-*co*-Glc copolymer films on flat silicon substrate and the concentrations of tripeptide DDD (Δ), SSS (○), VVV (□) and WWW (∇) solutions. The initial CA of the PNI-*co*-PT-*co*-Glc copolymer film is 92.2 ± 1.4 °. After being immersed in the solution of tripeptide and a following dry process by a flow of nitrogen gas, the film became much more hydrophilic. As shown in Fig. S7, relative hydrophobic WWW caused a larger decrease of CA than the moderately hydrophilic VVV, the hydrophilic DDD and SSS. This result was consistent with the wettability change on the copolymer film responding to tripeptide FFF, LLL and EEE, further demonstrating that the relative hydrophobic peptides composed of aromatic ring-bearing amino acids (e.g. FFF, WWW) induced much more obvious wettability change than the hydrophilic peptides. We presume that the aromatic rings might play a critical role in this process. All data are shown as mean ± s.d. (n=4-5).

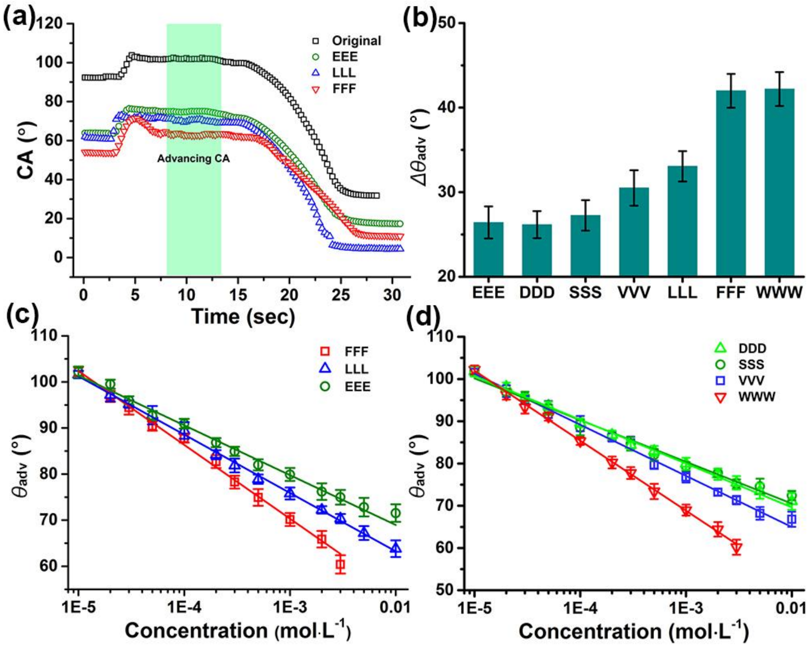

Figure S8. Peptide-selective dynamic wettability changes on the PNI-*co*-PT-*co*-Glc copolymer film in response to various tripeptides. (a) Time dependence of dynamic CA change of the original copolymer film (□) and the film treated by FFF (○), LLL (Δ), and EEE (∇) solution with a concentration of 3 mmol L^{-1}, respectively. These curves were recorded by goniometer during the process of expansion and contraction of a water droplet with a constant injection and withdrawal rate of about 1 μL s^{-1} in the range of 2 − 10 μL. The green part in the curve of Fig. S8a was used to calculate the advancing contact angle (θ_{adv}) through the software SCA20. (b) Comparison of the θ_{adv} decreases ($\Delta\theta_{adv}$) before and after the copolymer film was treated by different tripeptide solutions (3 mmol L^{-1}), respectively. (c, d) Relationships between the θ_{adv} and the concentrations of various tripeptide solutions. Due to the high water adhesion, receding contact angle (θ_{rec}) could not be measured accurately. Thus, the θ_{adv} was used to assess the dynamic wettability of the copolymer film responding to various tripeptides. Considering the significant difference in $\Delta\theta_{adv}$ of the copolymer film treated by different tripeptides, the hydrophobic aromatic peptides (e.g. FFF and WWW) caused much substantial changes in θ_{adv} compared with the hydrophilic peptides (e.g., EEE and SSS), which was highly consistent with the results of static CA experiments presented in the main text. All data are shown as mean ± s.d. (n=4-5).

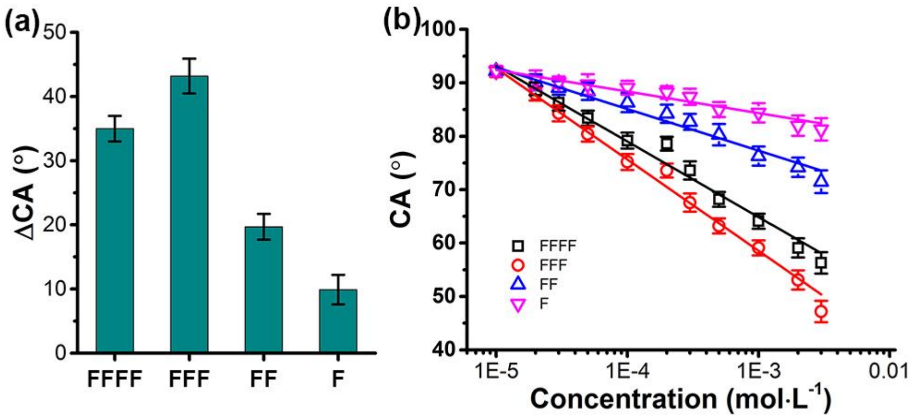

Figure S9. **A set of control experiments on static contact angle for tetrapeptide FFFF (Phe-Phe-Phe-Phe), dipeptide FF (Phe-Phe) and F (Phenylalanine).** (a) ΔCAs of the PNI-*co*-PT-*co*-Glc copolymer film on flat substrates treated by FFFF, FFF, FF and F solutions at an equal concentration of 3 mmol L^{-1}, respectively. (b) Relationship between CAs of the PNI-*co*-PT-*co*-Glc copolymer films on flat silicon substrate and the concentrations of FFFF (□), FFF (○), FF (Δ) and F (∇) solutions. The initial CA of the PNI-*co*-PT-*co*-Glc copolymer film is 92.2 ± 1.4°. After being immersed in the solution of various peptides and a following dry process by a flow of nitrogen gas, the film became much more hydrophilic. As shown in Fig. S9a, the ΔCA on the film upon the treatment of FFF solution was much larger than those of FFFF, FF and F solution, which was further confirmed by the dynamic relationship between CAs of the copolymer film and the concentrations of FFFF, FFF, FF and F solutions (Fig. S9b). All data are shown as mean ± s.d. (n=4~5).

Table S2. Comparison of surface static contact angles (CAs) on different polymer films on flat silicon substrate treated by tripeptide solutions (3 mmol·L^{-1}), respectively. P1: PNI-*co*-PT-*co*-Glc, P2: PNI-*co*-PT-*co*-AcGlc, P3: PNI-*co*-PT, P4: PNI-*co*-Glc, P5: PNIPAAm. All data are shown as mean ± s.d. (n=4~5).

Polymer film	Surface static contact angle [°]			
	Original	FFF treatment	LLL treatment	EEE treatment
P1	92.2 ± 1.4	47.4 ± 1.3	62.1 ± 1.3	70.7 ± 1.4
P2	95.1 ± 1.8	60.7 ± 1.3	71.3 ± 1.8	75.2 ± 1.6
P3	104.5 ± 1.2	81.5 ± 1.9	82.2 ± 1.2	81.0 ± 1.2
P4	64.7 ± 1.9	53.2 ± 1.2	58.3 ± 1.5	60.3 ± 2
P5	61.1 ± 2.0	59.3 ± 2.1	60.7 ± 1.2	60.1 ± 1.7

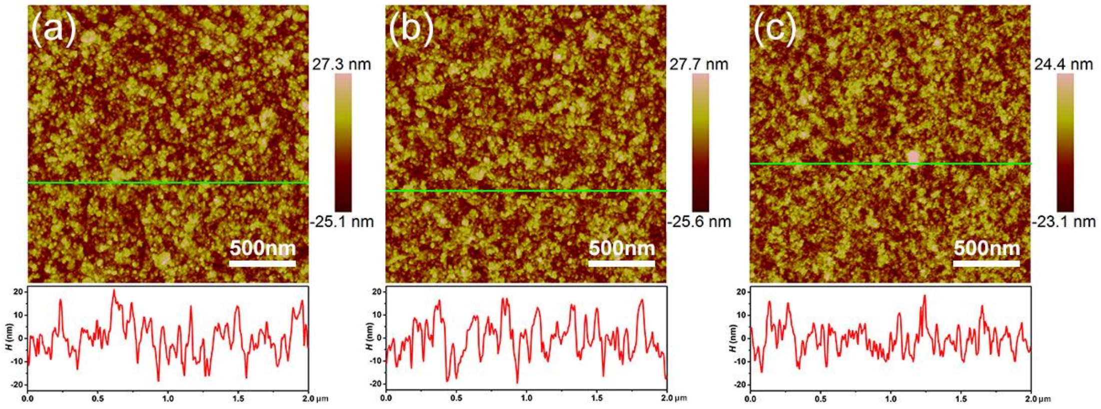

Figure S10. **AFM images of copolymer film and their section profiles.** AFM images of the PNI-*co*-PT-*co*-Glc copolymer film before (a) and after being treated with tripeptide EEE (b) and FFF (c), and their corresponding section profiles. Their surface roughness values were shown in **Table S3**. By comparing above AFM images, section profiles and surface roughness, we can find the copolymer brush film upon treatment by FFF changed from an original independent island-shaped structure (a) into a crosslinked structure (c), accompanied by reduction of surface roughness. However, no obvious change for the film treated by EEE was shown. This indicates adsorption of FFF into copolymer chain lead to a slight swelling for copolymer film.

Table S3. Surface roughness of the PNI-*co*-PT-*co*-Glc copolymer film before and after being treated with tripeptide EEE (PNI-*co*-PT-*co*-Glc + EEE) and FFF (PNI-*co*-PT-*co*-Glc +FFF).

	Arithmetic roughness (R_a) [nm][a]	RMS roughness (R_q) [nm][a]
PNI-*co*-PT-*co*-Glc	6.7	8.0
PNI-*co*-PT-*co*-Glc + EEE	6.6	7.9
PNI-*co*-PT-*co*-Glc + FFF	5.3	6.7

[a] These data were calculated from AFM height images by using NanoScope Analysis software (v1.40).

Table S4. Association constants (K_a) between various tripeptides and saccharide (Glc) or its acetylated derivative (AcGlc).

Guest tripeptides	K_a [L mol^{-1}][a,b]	
	Glc	AcGlc
DDD	$(8.72 \pm 0.63) \times 10^2$	$(6.51 \pm 0.32) \times 10^2$
EEE	$(3.95 \pm 0.12) \times 10^3$	$(1.57 \pm 0.83) \times 10^3$
SSS	$(8.41 \pm 0.91) \times 10^3$	$(3.77 \pm 0.89) \times 10^3$
VVV	$(5.45 \pm 0.12) \times 10^4$	$(5.03 \pm 0.31) \times 10^4$
LLL	$(3.54 \pm 0.28) \times 10^4$	$(2.90 \pm 0.20) \times 10^4$
FFF	$(9.87 \pm 0.50) \times 10^4$	$(8.98 \pm 0.59) \times 10^4$
WWW	$(1.18 \pm 0.12) \times 10^5$	$(1.01 \pm 1.17) \times 10^5$

[a] These data were calculated from the fluorescent intensity changes at 512 nm in Tris-buffer solution (1 mmol L^{-1}, pH 7.4) at 20 ℃.

[b] All error values were obtained by the results of nonlinear curve fitting, the correlation coefficient (R) of nonlinear curve fitting was over 0.99.

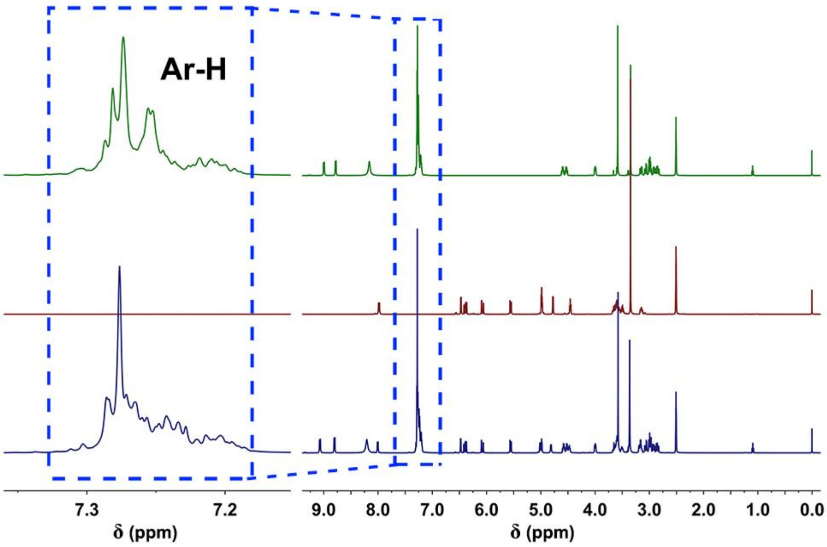

Figure S11. **Representative ^{1}H NMR spectra of FFF (green), Glc (red), and their 1:1 mixture (blue)**. Evident changes in the aromatic protons in FFF were observed upon the addition of FFF into saccharide unit Glc solution[5]. This result clearly described the detail on the participation of aromatic rings in the complexation between saccharide monomer Glc and the FFF.

References

[1] Tao, C. Z., Zhang, Z. T., Wu, J. W., Li, R. H. & Cao, Z. L. Synthesis of unnatural *N*-glycosyl α-amino acids via petasis reaction. *Chin. Chem. Lett.* **25**, 532–534 (2014).

[2] Peng, K. *et al.* Uniform, axial-orientation alignment of one-dimensional single-crystal silicon nanostructure arrays. *Angew. Chem. Int. Ed.* **44**, 2737–2742 (2005).

[3] Derjaguin, B. V., Muller, V. M. & Toropov, Y. P. Effect of Contact Deformations on the Adhesion of Particles. *J. Colloid Interface Sci.* **53**, 314–326 (1975).

[4] Babushok, V. I. & Zenkevich, I. G. Retention characteristics of peptides in RP-LC: peptide retention prediction. *Chromatographia* **72**, 781–797 (2010).

[5] Ferrand, Y., Crump, M. P. & Davis, A. P. A synthetic lectin analog for biomimetic disaccharide recognition. *Science* **318**, 619–622 (2007).
